# Overnight oximetry in children undergoing adenotonsillectomy: a single center experience

**DOI:** 10.1186/s40463-019-0391-2

**Published:** 2019-12-03

**Authors:** C. Carrie Liu, Kathleen H. Chaput, Valerie Kirk, Warren Yunker

**Affiliations:** 1 Sections of Otolaryngology - Head and Neck Surgery and Pediatric Surgery, Department of Surgery, Cumming School of Medicine, University of Calgary, Calgary, Alberta Canada; 2 Department of Obstetrics and Gynecology and Community Health Sciences, Cumming School of Medicine, University of Calgary, Calgary, Alberta Canada; 3 Division of Pediatric Respirology, Cumming School of Medicine, University of Calgary, Calgary, Alberta Canada

**Keywords:** Sleep oximetry, Obstructive sleep apnea, Sleep disordered breathing

## Abstract

**Background:**

Obstructive sleep apnea (OSA) is the most common indication for adenotonsillectomy in children. Home-based sleep oximetry continues to be used in the diagnosis of pediatric OSA despite a lack of correlation with lab-based polysomnography. This study investigates whether factors influence surgeons in selecting patients for home-based sleep oximetry, how the study findings are used in patient management, and whether abnormal oximetry findings are associated with post-operative complications.

**Methods:**

A retrospective review was performed on children with suspected OSA who had undergone a tonsillectomy and/or an adenoidectomy over a three-year period. Demographic features, comorbidities, pre-operative oximetry results, and post-operative complications were recorded. Data analysis consisting primarily of logistic regression was performed using Stata 12.0 (College Station, Texas).

**Results:**

Data was collected from 389 children. Two hundred and seventy-one children underwent pre-operative oximetry (69.7%). There was no significant association between age or the presence of comorbidities and the likelihood of undergoing pre-operative sleep oximetry. The post-operative complication rate was 0.8%. There was no significant association between abnormal sleep oximetry parameters and post-operative complications. Children with one or more abnormal sleep oximetry parameters were more likely to be observed in hospital for at least one night (OR 2.4, *p* < 0.0001).

**Conclusions:**

Our study suggests that surgeons are using home-based sleep oximetry findings to inform the post-operative care of children with suspected OSA, as those with abnormal home-based sleep oximetry findings were more likely to be observed in hospital. These hospital admissions may be unnecessary given the poor correlation of home-based oximetry and PSG as well as the low rate of serious post-operative complications.

## Introduction

Obstructive sleep apnea (OSA) has a prevalence of up to 10% in the pediatric population [1] and can result in neurocognitive and cardiovascular sequelae [2–5]. Polysomnography (PSG) is the gold standard for diagnosing OSA [6]. Despite their diagnostic value, lab-based polysomnography can be difficult to access [7]. A recent survey of the Canadian Pediatric Sleep Network showed significant geographical variance in the availability of PSG, with wait times ranging from less than 1 month to 2 years [8]. Furthermore, only 12% of children in Canada undergo polysomnography prior to adenotonsillectomy. The lack of accessibility to PSG has motivated health care providers to seek and investigate portable diagnostic means [8].

Lab-based overnight oximetry has been shown to have satisfactory correlation with PSG in the diagnosis of moderate to severe OSA [9]. The sensitivity and specificity of home-based overnight oximetry, however, is 67 and 60%, respectively, in the diagnosis of children with moderate OSA [10]. In contrast to lab-based oximetry, the interpretive internal algorithms that generate home-based oximetry reports are computerized and not manually validated, resulting in the potential incorporation of artifact into the final reports. Due to the poor agreement between home-based oximetry and PSG, the current recommendations do not support the use of this modality in the diagnosis of pediatric OSA [11].

Notwithstanding, our institution has observed the ongoing use of home-based sleep oximetry, both in public and private care settings. A review of the recent literature suggests that many clinicians are still using this modality as part of their standard approach to children referred for OSA [12–15]. In a survey of National Health Services otolaryngology departments in the UK, 84% of respondents reported using pre-operative pulse oximetry and among these practitioners, 58% used home-based sleep oximetry [15]. The continued use of home-based sleep oximetry likely reflects the lack of access to PSG that is seen in many care settings, predominately in publicly funded health systems. It may, however, also reflect a lack of awareness regarding current recommendations.

Given the continued use of home-based sleep oximetry in many institutions, it is important to investigate how this modality is being used and its effects on patient care. The current study investigates the experience with home-based overnight sleep oximetry at our institution. Specifically, we investigated the factors that influence practitioners in ordering home-based oximetry. We also examined whether sleep oximetry findings affect patient management in terms of overnight admission versus day surgery. Finally, we investigated whether specific abnormal oximetry findings are associated with peri-operative complications.

## Methods

This was a retrospective review of children who underwent tonsillectomy and/or adenoidectomy surgery at the Alberta Children’s Hospital during the period of January 1, 2005 to December 31, 2009. Approval for the study was obtained from the Conjoint Health Research Ethics Board at the University of Calgary.

### Patient selection

The study population consists of a series of children undergoing adenotonsillectomy, tonsillectomy, or adenoidectomy surgery at the Alberta Children’s Hospital. Subjects were identified using a computerized search and were included if the following criteria were met: children aged 1–18 years who underwent adenotonsillectomy, adenoidectomy, or tonsillectomy, and with a suspected diagnosis of SDB/OSA. Children were excluded if they had a history of prior airway surgery (i.e. cleft palate repair, uvulopalatoplasty, adenoidectomy and/or tonsillectomy).

### Data collection

Hospital charts were reviewed and pertinent data regarding demographic information, comorbidities, pre-operative oximetry results, surgery performed, and peri-operative complications were recorded. Serious complications included death, cardiorespiratory arrest, acute respiratory distress, and unplanned pediatric intensive care admissions. The length of hospitalization was recorded. At our institution, children are admitted after adenotonsillectomy if they are younger than 3 years of age, have severe OSA as defined by AHI ≥ 10 or oxygen saturation nadir < 80%, or if they have specific comorbid conditions (ie. trisomy 21, neuromuscular disease, craniofacial abnormalities) [16]. Children may also be admitted if there are post-operative concerns such as poor oral intake, supplemental oxygen requirements, or uncontrolled pain. An unnecessary admission is defined as an admission outside of these parameters.

Oximetry data generated by pre-operative home studies were recorded. All home-based oximetry studies were performed using the N595 Nellcor oximeter (Medtronic). The averaging time was set at the fast mode (2–3 s). As defined by Nixon et al., a “desaturation” was defined as a decrease in oxygen saturation of ≥4% and a “cluster of desaturations” was scored as ≥5 desaturations occurring in a 10- to 30-min period [17]. Mean and minimum oxygen saturation, time spent below 90% and time spent below 80% was also recorded.

There are validated scoring systems for lab-based sleep oximetry that record the number of desaturations below a certain percentage [17]. The respirology team at our institution chose a modified scoring system to interpret and classify overnight oximetry results. The McGill scoring system was based on data obtained using an N200 oximetry unit [17]. This unit allows for the visualization of the pulse waveform, which a measure of signal integrity. The oximetry devices at the Alberta Children’s Hospital do not collect pulse waveform data. As such, we used a three-scale system that blended the McGill classifications of two and three, with the intention of avoiding overinterpretation or under-interpretation of the oximetry findings. The oximetry outcome groups were 1 – normal (no desaturations), 2 – abnormal (desaturations which did not meet criteria for diagnosis of OSA), 3 – findings consistent with OSA (clusters of desaturations occurring regularly throughout the recording).

### Data analysis

Demographic data was summarized using descriptive statistics. Logistic regression was performed to examine the factors that contribute to ordering oximetry, the association between abnormal oximetry findings and complications rates, and whether abnormal oximetry findings were associated with an increased length of stay in hospital. All analyses were performed using STATA 12.0 (College Station, Texas).

## Results

A total of 389 children were included. Table 1 summarizes the demographic information of the study subjects. In total, home-based overnight sleep oximetry was performed in 271 (69.7%) children with suspected OSA/SDB undergoing surgery. Thirty children (7.7%) had a chromosomal abnormality, metabolic syndrome, or cardiac condition. There was no association between age or comorbidities and the likelihood of undergoing pre-operative sleep oximetry (OR 1.0, *p* > 0.05 and 0.9, p > 0.05, respectively).

**Table 1:** Study subject characteristics

	Total (*n* = 389)	Adenotonsillectomy (*n* = 271)	Adenoidectomy (*n* = 95)	Tonsillectomy (*n* = 23)
Age in years (SD)	5.8 (3.3)	6.0 (3.2)	4.7 (2.8)	8.7 (4.6)
Males (%)	214 (55%)	148 (55%)	54 (57%)	12 (52%)
Pre-op oximetry performed (%)	271 (70%)	191 (70%)	64 (67%)	16 (70%)

Of 389 operations, there were a total of 3 (0.8%) serious complications, with 2 resulting in intensive care unit (ICU) admissions (Tables 2 and 3). One child had post-obstructive desaturations secondary to aspiration pneumonia, which improved with conservative management with intravenous antibiotics and incentive spirometry on the inpatient ward. The child was discharged home after a 5-day admission and was deemed by the pediatrics inpatient team to not require outpatient follow-up from a pulmonary perspective. The other two children had significant obstructive events post-operatively, with prolonged apneic spells and oxygen desaturations to below 70%. Both children required admission to the ICU for administration of oxygen via face mask. They were discharged 3 and 7 days later with community follow-up with a pediatrician. We observed no significant association between abnormal sleep oximetry parameters and post-operative complications (Table 4).

**Table 2:** Serious post-operative complications

Patient	Age (years)	Comorbidities	Surgery performed
1	8	No	AT
2	2	No	AT
3	1	No	A

*AT* adenotonsillectomy, *A* adenoidectomy

**Table 3:** Serious post-operative complications and the corresponding oximetry findings

Patient	Mean SpO_2_	Min SpO_2_	% time with SpO_2_ < 90%	% time with SpO_2_ < 80%	Clusters
1	96%	89%	1%	0%	No
2	88%	58%	63%	2%	Yes
3	96%	76%	2%	0.1%	Yes

Min SpO_2_, minimum oxygen saturation; clusters defined by ≥5 desaturations ≥4% in a 10–30-min period

**Table 4:** Complications and abnormal oximetry parameters

Parameter	Odds ratio (95% CI)	*p*-value
Nadir < 90%	1.0 (0.1, 11.4)	0.9
Nadir < 80%	7.0 (0.6, 77.9)	0.1
SpO_2_ < 90% ≥1 min	3.5 (0.3, 38.7)	0.3
SpO_2_ < 80% ≥1 min	8.3 (0.7, 94.8)	0.2
Clusters of desaturations	3.6 (0.3, 39.9)	0.3
≥1 of the above parameters	2.1 (0.2, 23.2)	0.6

Two hundred and nineteen children (56.3%) were observed in hospital for at least one night, while the remainder had same-day discharge. Of those with admissions, 57 were admitted for age under 3, a comorbidity that warranted admission, or an oxygen saturation nadir of < 80%. One (0.5%) child was admitted for post-operative fevers. The remaining 162 admissions did not have clear indications and are considered unnecessary.

One hundred and ninety-one children (49.1%) had one or more abnormal oximetry parameter (Table 5). When controlled for age, those with at least one abnormal sleep oximetry parameter had a 2.4 increase in the odds of having an unnecessary admission, compared to children with normal parameters (*p* < 0.0001). Specifically, children who had oxygen nadirs < 90% and clusters of desaturations on oximetry had 2.4 and 2.1 times the odds of unnecessary admissions, respectively (p < 0.0001 for both). The proportion of children in each oximetry outcome group who had an unnecessary admission is shown in Table 6.

**Table 5:** Abnormal home-based sleep oximetry results

Abnormality	No. of children (%)
Nadir < 90%	128 (33%)
Nadir < 80%	88 (23%)
SpO_2_ < 90% ≥1 min	143 (37%)
SpO_2_ < 80% ≥1 min	23 (6%)
Clusters of desaturations	140 (36%)

**Table 6:** Proportion of children who had unnecessary admissions in each oximetry outcome group

Oximetry outcome group	No. of patients with finding	No. of patients with admission (%)
1 (normal oximetry)	198	62 (31.3%)
2 (desaturations not meeting criteria for OSA)	128	72 (56.3)
3 (clusters of desaturations)	140	75 (53.6%)

## Discussion

Currently, the use of home-based studies is not recommended in the diagnosis of pediatric OSA [11]; however, there remains utilization of home-based sleep oximetry, possibility as a result of the resource scarcity that is seen in many care settings [14, 15]. Our study showed that there is variability with which home-based sleep oximetry is ordered and interpreted among surgeons. In our cohort, 30.3% of children with suspected OSA/SDB did not undergo pre-operative oximetry. This likely reflects practice variation among surgeons; some surgeons diagnose and treat OSA on the basis of the clinical history and physical exam, whereby others defer diagnosis and treatment until after the overnight sleep oximetry study is performed. Surgeons may also be using sleep oximetry as a means to triage and determine the urgency of surgery. Finally, our study suggests that surgeons may be using oximetry to inform perioperative patient management, as those with abnormal oximetry findings were more likely to be observed in hospital overnight.

The association between abnormal oximetry findings and increased post-operative observation suggests that physicians are using oximetry as a way to estimate the risk of post-operative complications. Overall, we found a very low rate of severe post-operative complications (0.8%). This is consistent with the complication rates reported in the literature [18]. We did not find a significant association between any abnormal sleep oximetry parameters and the occurrence of post-operative complications.

Our study contributes to the existing literature in that there appears to be no utility in the use of home-based sleep oximetry, especially as a method of predicting post-operative complications. Furthermore, given the finding that children with abnormal sleep oximetry studies have a higher odd of being admitted, along with the low overall rate of post-operative complications, home-based sleep oximetry and the admissions based on oximetry findings may represent an unnecessary cost to the health care system.

The main limitation of this study is the low number of peri-operative serious complications. As such, our study was not sufficiently powered to analyze the association between respiratory complications and certain sleep oximetry findings. Fortunately, modern surgical and anesthetic techniques are associated with low rates of post-adenotonsillectomy complications. Future studies with larger sample sizes and greater numbers of complication outcomes are required. A second and important limitation of our study is that the sleep oximeter used at our center does not allow for the visualization of the pulse waveform. Visualization of the pulse waveform is important as it allows for the determination of signal integrity and distinction of true desaturations. Unfortunately, modern oximetry units have internal algorithms that delete poor data automatically, precluding the clinician’s ability to examine the raw data for true desaturations. As a result, we were unable to use a validated scoring system such as the McGill Score for oximetry interpretation [17]. A third limitation of this study is the risk for selection bias given its retrospective design. The study cohort is from a tertiary pediatric hospital, where children who are referred to otolaryngologists for surgery may have been deemed by the referring doctor to have more clinically significant OSA based on the history and physical exam. It is possible that these children were more likely to be admitted based on the clinical history alone. However, our finding is of the association between abnormal sleep oximetry parameters and unnecessary admissions. As there is no known correlation between clinical history and home-based sleep oximetry results, our finding of an increased odds of admission based on abnormal sleep oximetry parameters more likely to reflect the surgeon’s interpretation of the oximetry results rather than their evaluation of the patient’s clinical history.

## Conclusion

Our study examined the practice patterns surrounding overnight home-based sleep oximetry. We found that surgeons are using sleep oximetry to determine post-operative patient care, as children with abnormal sleep oximetry findings were more likely to be observed in hospital overnight. The ongoing use of home-based sleep oximetry and the hospital admissions that stem from abnormal sleep oximetry findings may represent a waste to the health care system.

## Data Availability

The datasets used and/or analyzed during the current study are available from the corresponding author on reasonable request.
